# Family history in breast cancer in São Luís, Maranhão, Brazil

**DOI:** 10.1186/s13104-015-1471-7

**Published:** 2016-03-10

**Authors:** Maria Hilda Araújo Ribeiro, Marcos Antonio Custódio Neto da Silva, Walbert Edson Muniz Filho, Anna Cyntia Brandão Nascimento, Rodrigo Duart Martins Souza, Carlos Eduardo Everton Machado, Dulcelena Ferreira Silva, Geusa Felipa de Barros Bezerra, Graça Maria de Castro Viana, Maria do Desterro Soares Brandão Nascimento

**Affiliations:** Medicine Course, State University of Maranhão, Caxias, Maranhão Brazil; Medicine Course, Federal University of Maranhão, São Luís, Maranhão Brazil; Department of Pathology, Nucleum of Basic and Applied Immunology, São Luís, Maranhão Brazil; University Hospital of Federal University of Maranhão, São Luís, Maranhão Brazil; Department of Morphology, Federal University of Maranhão, São Luís, Maranhão Brazil; Postgraduation Program in Adult and Child Health, Department of Pathology, Nucleum of Basic and Applied Immunology , São Luís, Maranhão Brazil; Rua de Santa Aninha, no 68, Centro, São Luís, MA CEP 65010-320 Brazil

**Keywords:** Family history, Breast cancer, Epidemiology

## Abstract

**Background:**

Familial cancer includes some types of cancer aggregation without a well-defined inheritance pattern. Cancer genetics is an essential component of clinical practice in oncology. In Brazil, breast cancer is the leading cause of death in women. In Maranhão, studies on genetic predisposition are necessary to investigate the incidence and mortality rates. The aim of this study was to investigate familial cancer among relatives of women who died of breast cancer in São Luís, Brazil, constructing a pedigree to identify families with a hereditary predisposition, an important step in the early diagnosis of malignant tumors.

**Methods:**

The city of São Luís is located on the Island of Maranhão, northeastern Brazil, with a population of 997,098 inhabitants mainly comprising blacks and mulattoes, including descendants of runaway slaves from the Amazon region itself. Data for pedigree construction were obtained from the records of 54 patients seen at the Aldenora Bello Institute of Oncology, São Luís, between 2000 and 2007, as well as by interview with relatives of the patients.

**Results:**

The mean patient age at diagnosis was 39.5 years. Most women were mulattoes (36/54, 66.6 %). A history of cancer was observed in 18 families, with 16 families possessing cases of cancer among first-degree relatives and five among second-degree relatives.

**Conclusion:**

A concentration of cancer cases was found in families of patients diagnosed until the age of 40, a finding demonstrating the importance of a family history prior to genetic counseling.

## Background

Breast cancer is the second most frequent type of cancer in the world and the most common among women. The highest rates are observed in North America and in South American countries such as Brazil and Argentina [1]. In Brazil, estimates for 2014 indicated 57,120 new cases of breast cancer, including 570 in the State of Maranhão and 250 of them in the municipality of São Luís [2].

Data from the Brazilian Institute of Geography and Statistics (IBGE) showed that life expectancy at birth in Brazil increased from 74.6 to 74.9 years in 2014. The State of Maranhão has a life expectancy of 69.7 years; 66 years for men and 73.7 years for women. In Maranhão, access to health services is still difficult, because of the low economic status of the population and the lack of public policies for the organization of health care [3].

Hereditary cancer comprises diverse syndromes that are relatively rare and of monogenic etiology. These syndromes are estimated to correspond to about 5–10 % of cancer cases in the general population [4, 5]. An elevated risk of breast cancer in first- and second-degree relatives of patients with an apparently sporadic cancer has been reported in various population studies [4, 6–8]. Genetic factors predisposing to hereditary cancer are believed to be responsible for 5.0–7.0 % of all cases of breast cancer. Hereditary cancer syndromes lead to the occurrence of disease at a younger age than that of the population with sporadic breast cancer, with approximately 25.0 % of cases being diagnosed before the age of 40 [9, 10]. Other studies have reported an increased risk of breast cancer among individuals younger than 50 and 60 years, respectively [6, 11]. Women who inherit a loss-of-function mutation in one of the alleles of the BRCA1 or BRCA2 genes present a 85.0 % higher risk of developing breast cancer until the age of 70 [11, 12].

Analysis of cancer family history may reveal the existence of various other cases of disease with peculiar characteristics, such as relatives affected in three successive generations, two or more first-degree relatives with a premenopausal diagnosis of the disease, cases of bilateral breast cancer, and cases of breast cancer in men [13].

National Institute for Health and Clinical Excellence (NICE) guidelines recommend the use of information from family history, which should be taken into account in assessing risk and deciding whether and when to refer, and when to reassure [14]. Women with increased risk based on a positive family history as described within NICE guidelines may be advised to undergo a range of different forms of surveillance, genetic testing or even preventive management [15].

The definition of the form of tracking high-risk women have not yet supported on current scientific evidence and is varied to approach this group in national screening programs. It is recommended that women at high risk of breast cancer have individualized clinical monitoring. The National Institute of Cancer of Brazil (INCA) recommends screening for women at high risk of breast cancer, whose routine should start at age 35 with breast examination and annual mammography [16].

With respect to the histopathological classification of breast tumors, a predominance of infiltrating ductal carcinoma is observed, which accounts for 65.0–85.0 % of these malignant tumors. However, the relationship between histological type and hereditary cancer is still not well established [17–19].

Family health history has long been acknowledged as an important part of the medical examination [20]. In breast cancer, family history is a key risk factor of breast cancer [21–23]. Women with a strong family history of breast cancer could inherit genetic alterations that modify their risk of disease [24]. Study conducted in Spain showed that 20 % of invasive breast carcinomas had a family history of cancer [25].

Primary care has an important role in the prevention and early diagnosis of hereditary cancer. Reduction in risk through prevention is a core function of primary care [26].

In Brazil, genetic tests are still not available through the public health service and the construction of pedigrees is therefore important for the investigation of cases of cancer among relatives. The possibility of identifying relatives who are at high risk for the development of cancer permits the application of preventive measures and the early detection of cancer. Patients classified as high risk according to cancer family history could be referred for genetic counseling at other centers of the country.

In view of the lack of studies on this subject in the State of Maranhão, the objective of the present investigation was to evaluate the occurrence of malignant tumors in first- and second-degree relatives of patients with a diagnosis of breast cancer who died before the age of 60.

## Methods

An observational, descriptive study divided into two phases was conducted: a retrospective study reviewing the records of 54 patients from São Luís who had died of breast cancer before the age of 60 and had a histopathological exam revealing a malignant breast tumor and a prospective study interviewing relatives for the collection of information about cancer history in these families. Since according to the literature, hereditary cancer syndromes, especially Li-Fraumeni syndrome, affect individuals with one first- or second-degree relative with a typical tumor of this syndrome identified at any age and with another first- or second-degree relative with cancer diagnosed before the age of 60 [27]. Due to this information, this age was adopted as an inclusion criterion.

The study involved 54 women who lived at São Luís—MA seen at the Aldenora Bello Maranhense Institute of Oncology (IMOAB) between 2000 and 2007. This service is certified as a Reference Center of High Complexity in Oncology (CACON). In the analyzed period, there were 158 deaths due to breast cancer, but were recruited only women’s medical records residing in São Luís, so only 54 were included. This is a convenience sample from the 158 recorded deaths. Because of the difficulty of recruiting relatives of women who resided in the State of Maranhão, only 54 women were included because they lived in São Luis. Family members were invited to participate in the study and after signing an informed consent were included. All family members agreed to participate in the study and there was no loss to follow-up during the period.

The following characteristics of morbidity and mortality were analyzed: age at diagnosis and death, overall survival, tumor laterality, tumor stage, associated tumors, and presence of metastases. The presence of other cancer cases in the family, number of affected relatives, degree of kinship, and other types of malignant tumors observed in the family until the third generation were also investigated. The recommendations of the National Comprehensive Cancer Network and of the project Guidelines for Hereditary Cancer were used as criteria of hereditary cancer [4, 27]. Parents, siblings and sons/daughters of index cases were defined as first-degree relatives, and uncles/aunts and grandparents were defined as second-degree relatives [28].

Sociodemographic data, histopathological type, handedness when it comes to breast cancer, staging, overall survival and site-metastatic were obtained by review of medical records.

The study was approved by the Research Ethics Committee of the University Hospital, Federal University of Maranhão (No. 362/07). The Statement of Informed Consent Form (ICF) was presented to the families of women who died from breast cancer and signed in obedience for Resolution No. 196/96 and its complementary. The information contained in pedigrees obtained maintains the confidentiality of the study patients and their families.

The data were tabulated using the Microsoft Excel 2003 program and analyzed with the Epi-Info 2007 program, version 3.4.3.

## Results

A total of 158 deaths due to breast cancer were observed among women aged 60 years or younger between 2000 and 2007. Fifty-four women with breast cancer who lived in São Luís—MA were studied.

The age of these women at the diagnosis ranged from a minimum of 22 years to a maximum of 60. The mean age was 39 years and 6 months, with a standard deviation of 7.52 and the average (age of) death of 41.98 years, with a standard deviation of 7.91 age. The demographic data presented in Table 1 show that the vast majority of women was of mixed race (n = 36/54, 66.6 %), married (or in a stable relationship) (n = 29/54, 53.7 %). Most women (30) studied had reasonable education (secondary or higher) (n = 30/54, 55.6 %). It was observed that most diagnoses of breast cancer was performed in young women aged 30–39 years (55.8 %).

**Table 1:** Sociodemographic characteristics of women who died by breast cancer, IMOAB, 2000–2007, São Luís—MA

Variables	Frequency	Percent
Age in years		
20–29	4	7.4
30–39	30	55.8
40–49	14	25.9
50–59	5	9.3
60	1	1.9
Ethnicity		
Black	15	27.7
White	2	3.7
Brown	36	66.6
Not declared	1	1.9
Marital status		
Single	21	38.8
Married or stable union	29	53.7
Widowed	0	0.0
Separated	3	5.5
Not declared	1	1.9
Education		
No school	1	1.9
Uncompleted 1° school	18	33.3
1° school only	5	9.2
Uncompleted 2° school	0	0.0
Completed secondary school	23	42.6
Uncompleted college	1	1.9
College	6	11.1

The most common histological type was infiltrating ductal carcinoma (83.3 %), followed by the association of infiltrating ductal carcinoma/infiltrating lobular carcinoma (7.4 %). One patient (1.9 %) had lobular carcinoma and another (1.9 %) had comedocarcinoma. The right breast was the most affected (24/54, 44.4 %). The left breast was affected in 21 (38.8 %) patients and 7 (12.9 %) presented a bilateral tumor. In the cases studied, there were no cases of family history in bilateral cancer, with no information being available for two (3.7 %) patients. Lymph node involvement was observed in 36 (66.7 %) women and all of them developed distant metastases, except for one patient. The most frequent metastatic sites were bone (23/54, 42.6 %), lungs (19/54, 35.2 %), and liver (15/54, 27.8 %) (Table 2).

**Table 2:** Clinical characteristics of women who died from breast cancer. IMOAB, 2000–2007, Sao Luis—MA

Clinical characteristics	f	%
Family history of breast cancer		
No	29	53.7
Yes	18	33.3
Not declared	7	13.0
Tumor localization		
Right	24	44.4
Left	21	38.9
Bilateral	7	13.0
Not declared	2	3.7
Histological type		
Invasive ductal carcinoma	45	83.3
Invasive ductal and lobular carcinoma	4	7.4
Carcinoma SOE	2	3.7
Lobular carcinoma	1	1.9
Invasive ductal carcinoma associated to Paget’s disease	1	1.9
Comedocarcinoma SOE	1	1.9
Cancer stage		
II	13	24.0
III	27	50
IV	14	26.0
Lymph node invasion		
Yes	36	66.7
No	16	29.6
Not declared	2	3.7
Metastasis		
Bone	23	42.6
Lung	19	35.2
Liver	15	27.8
Brain	11	20.4
Pleura	4	7.4
Others	4	7.4

With respect to tumor stage, there was a predominance of stage III among the 54 women studied (n = 27; 50.0 %). Stage II was observed in 13 (24.0 %) women and stage IV in 14 (25.93 %) (Table 2). Furthermore, 56.1 % of the patients with stage III and IV cancer were between 30 and 39 years of age at diagnosis and 5.6 % of the women with stages III and IV were 29 years or younger (Table 3). Eighteen (33.3 %) of the 54 families presented a history of cancer (Table 3). Only one case of cancer, in addition to the index case, was observed in 13 families (72.2 %). Three other families (16.6 %) reported two cases of cancer. Among patients with a family history of cancer, 61.1 % were diagnosed at age 39 or younger and only 22.2 % (4/18) of these patients were 50 years or older. The malignant tumors identified in the families included lung cancer in eight (27.4 %), ovary cancer in four (prostate cancer in three (13.7 %), ovary cancer in four and melanoma in three (10.3 %).

**Table 3:** Distribution of women who died of breast cancer and relationship with a family history of cancer and stage (IMOAB, São Luís, 2000–2007)

Age range (years)	Family history of cancer						Stage					
	No		Yes		Unknown		II		III		IV	
	N	%	N	%	n	%	n	%	N	%	n	%
20–29	1	3.5	2	11.1	1	14.3	1	7.7	2	7.4	1	7.1
30–39	17	58.6	9	50.0	4	57.1	7	53.8	16	59.3	7	50.0
40–49	9	31.0	3	16.7	2	28.6	4	30.8	6	22.2	4	28.7
50–59	2	6.9	3	16.7	0	0.0	1	7.7	3	11.1	1	7.1
60	0	0.0	1	5.6	0	0.0	0	0.0	0	0.0	1	7.1
Total	29	100.0	18	100.0	7	100.0	13	100.0	27	100.0	14	100.0

In this study, overall survival ranged from 1 to 240 months, with an average of 30.2 months and a median of 16 months, and the 5-year survival was 85.1 %. It is also observed that 11 women with stage II survived two to 48 months (5 years) and only one had to 240 months. A relationship was observed between tumor stage and overall survival, with 85.7 % of the patients with initial tumor stage IV and 22.2 % with stage III presenting survival less than 12 months (Table 4).

**Table 4:** Distribution of women seen at IMOAB who died of breast cancer according to tumor stage and its relationship with survival (São Luís, 2000–2007) (n = 54)

Stage	Survival (months)										
	1–12	13–24	25–36	37–48	49–60	61–72	73–84	85–96	97–156	157–240	Total
II	2	5	1	3	0	0	0	0	1	1	13
III	6	12	1	3	0	2	2	1	0	0	27
IV	12	0	0	1	0	1	0	0	0	0	14
Total	20	17	2	7	0	3	2	1	1	1	54

Analysis of first-degree relatives demonstrated the occurrence of melanoma in one family involving two first-degree relatives, one of them also having a diagnosis of breast cancer. Two cases of cancer (prostate cancer and melanoma) were observed in another index case. Malignant tumors also occurred sporadically in second-degree relatives, with the observation of lung cancer in two families and a case of prostate cancer in another. The distribution of multiple cases of cancer is shown in Table 5. 10 (71.4 %) families presented two or more cases along two generations, three (21.4 %) had two or more cases in the same generation, and one reported three cases in three generations. Sixteen (37.2 %) families reported cases among first-degree relatives of the index case and five (11.6 %) among second-degree relatives. Among families with a history of cancer, 10 (71.4 %) reported cancer in only first-degree relatives, three (21.4 %) exclusively in second-degree relatives, and one family had cases among first- and second-degree relatives.

**Table 5:** Distribution of cases of cancer in relatives of women diagnosed with breast cancer in IMOAB. St. Louis, 2000–2007

Index case	Stage	Family														
		1° and 2° degrees														
		Lung		Prostate		Melanoma	Ovary		Breast		Colorectal	Stomach	Pancreas	Larynx	Esophagus	Uterine cervix
		1°	2°	1°	2°	1°	1°	2°	1°	2°	1°	1°	1°	1°	2°	2°
1°	IV	0	0	0	0	0	1	0	0	0	0	0	0	0	0	0
2°	IV	0	1	0	0	0	0	0	0	0	0	0	0	0	0	0
3°	IV	0	0	0	1	0	0	0	0	0	0	0	0	0	0	0
4°	IV	0	0	1	0	1	0	0	0	0	0	0	0	0	0	0
5°	IV	1	0	0	0	0	0	0	0	0	0	0	0	0	0	0
6°	IV	1	0	0	0	0	0	0	0	0	0	0	0	0	0	0
7°	IIa	0	0	1	0	0	0	0	0	0	0	0	0	0	0	0
8°	IIIb	0	0	0	0	0	1	0	0	0	0	0	0	0	0	0
9°	IIIb	0	0	0	0	0	0	0	0	0	0	1	0	0	0	0
10°	IIIc	0	0	0	0	0	0	0	0	0	0	0	1	0	0	0
11°	IIa	1	1	0	0	0	0	0	0	0	0	0	0	0	0	0
12°	IIa	0	0	0	0	2^a^	0	0	1^a^	0	0	0	0	0	0	0
13°	IIa	0	0	0	0	0	0	0	0	0	1	0	0	0	0	0
14°	IIIb	1	0	0	0	0	0	0	0	0	0	0	0	0	0	1
15°	IV	0	0	0	0	0	0	1	0	0	1	1	0	1	1	0
16°	III	0	0	0	0	0	0	0	0	0	0	1	0	0	0	0
17°	IV	1	0	0	0	0	0	0	0	0	0	0	0	0	0	0
18°	II	0	1	0	0	0	1	0	0	2	0	0	0	0	0	1
		5	3	2	1	3	3	1	1	2	2	3	1	1	1	2

^a^In the same family of the index case was diagnosed cancer in breast and skin

**Fig. 1:**
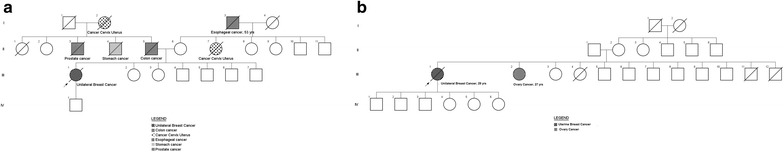
**a** Pedigree of a family with breast cancer history and association with colorectal cancer. **b** Pedigree of a family with breast cancer history and association with ovarian cancer. Male family members are represented by a square and female by a circle while a line indicates the family member is deceased

**Fig. 2:**
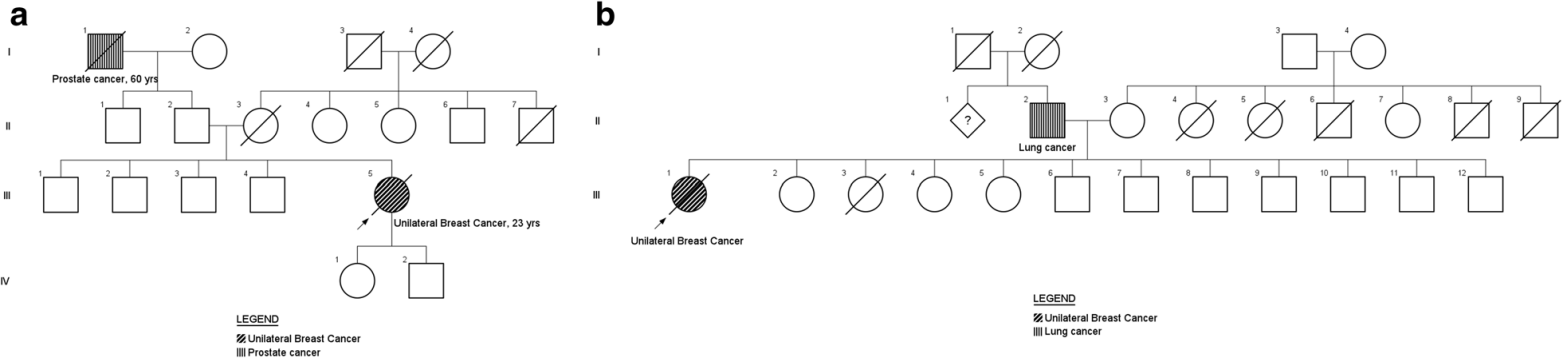
**a** Family history of prostate cancer. **b** Family history of lung cancer. Male family members are represented by a square and female by a circle while a line indicates the family member is deceased

An association of breast and ovarian cancer (breast-ovarian syndrome) was observed in two women (11.1 %, 2/18) and association of breast and colorectal cancer (breast, colorectal syndrome) in a woman (5.5 %, 1/18), and other cases (83.3 %, 15/18). Figure 1 shows pedigrees of families with breast cancer history and association with colorectal cancer (1a) and ovarian cancer (1b). In the represented case, the increase is evident in the risk of malignancies in patients with these syndromes. Of the 18 families who reported a history for cancer, two are represented by pedigrees described in Fig. 2. Figure 2a is a case of family history of prostate cancer, which is the most prevalent tumor in men in Brazil. Figure 2b shows a case of family history of lung cancer, which is a high morbidity and mortality tumor.

## Discussion

Breast cancer mortality rates continue to be high in Brazil, because in most cases the diagnosis is made during a late stage of the disease. According to Abreu and Koifman [17], stages III and IV correspond to about 60 % of all initial diagnoses. Even higher rates were observed in the present study, with 75.9 % of the patients presenting stage III and IV cancer. Of these, 5.5 % were women aged 29 years or younger, a finding that might be explained by the fact that there is no routine indication of mammography for women of this age, with a consequent delay in diagnosis.

Variations in the incidence of breast cancer among young women are reported in the literature. In the study of Silva [19], 22.3 % of the patients were younger than 40 years. Oliveira et al. [29] observed 5.5 % of patients aged 35 years or younger and 36.4 % of them reported other cases of cancer in the family. In the present study, 4/54 (7.4 %) of the patients were diagnosed before the age of 29 and 34/54 (62.9 %) before the age of 39. The percentage of patients younger than 30 years (7 %) observed here was lower than that reported in the study of Oliveira et al. [29] in which this age group accounted for 20.0 % of cancer cases.

Eisenberg [30], following up women with infiltrating ductal carcinoma younger than 60 years in the State of Rio de Janeiro for 60 months, found a survival of 55 months. This finding is in contrast to the present investigation [30]. However, deaths that occurred after the 60 months of follow-up were not included in that study, a fact that might explain this difference. In agreement with the present study, Silva [19] observed a mean survival of 39.3 months among women who died of breast cancer. The lack of recommendation of mammography screening for this age group may explain the late diagnosis in young women. Therefore, it is important to obtain a family history of cancer during anamnesis.

Several studies have demonstrated a relationship between breast cancer and other cancer cases in the family, with the prevalence of cancer cases ranging from 5 to 10.0 % [8, 9, 13, 31, 32]. The prevalence of a family history of cancer was high in the present study (33.3 %) with a predominance of sporadic cases.

In studies designed to evaluate the relationship between a certain type of cancer and a family history of this disease, some confounding factors may impair the analysis such as a small number of affected subjects and difficulties in the confirmation of family history [4]. In the present study, most families with a history of cancer, including the index cases, presented at least two cases of malignant neoplasms. However, confirmation of the tumor was not possible in most cases, a fact impairing a more accurate analysis of the data collected. On the other hand, considering only families with three or more cases, the frequency obtained was similar to that reported in the literature.

In the study of Vieira [9], the tumors observed in families of index cases included colon, rectal and prostate cancer, melanoma, and hepatic carcinoma. In the present study, the most frequent tumors were lung cancer, prostate cancer, melanoma, and ovarian cancer [9]. Lung cancer was the most frequent in these families. However, the strong environmental component involved in the genesis of this tumor and the impossibility to investigate the life habits of these cases did not permit to establish a relationship with hereditary cancer [33].

Two index cases call attention because of the particularities of their evolution. The first was an association with pregnancy in a 29-year-old woman. According to the patient, nodules arose in her right breast by the sixth month of gestation but no appropriate investigation was performed. During the puerperium, after treatment for mastitis without an adequate response, the diagnosis of a malignant breast neoplasm was established. However, distant disseminated tumor cells were detected and the patient died after only 2 months. The family history of this index case showed a case of cervical cancer.

Breast cancer is the second most frequent tumor associated with pregnancy and hypertrophy and breast engorgement during this period may contribute to a delay in diagnosis. However, the disease itself does not indicate a poor prognosis. In a series of 15 cases of pregnancy-associated breast cancer most patients had locally advanced disease at diagnosis but progressed satisfactorily and were free of the disease [34]. We found no study in the literature investigating the relationship between breast cancer during pregnancy and hereditary cancer syndromes.

Another woman aged 50 years at the time of diagnosis of breast cancer presented a history of melanoma. The patient had been treated but recurrence was observed 1 year later, after the diagnosis of breast cancer had been made. The stage of the breast tumor was IIa and the patient died at 52 years, with survival of 25 months. Only lymph node involvement was demonstrated.

Limitations of this study are the small number of included cases, large account of the women reside in the interior of state of Maranhão; Absence of a control group of to compare if there was a greater risk of family cancer in women who have had breast cancer.

## Conclusions

In conclusion, the evaluation of family history of breast cancer by pedigree construction permitted the identification of family groups with an elevated risk of familial cancer. This finding demonstrates the relevance of this strategy for primary prevention and early detection of breast cancer in the Maranhão population.

## Abbreviations

BRCA1: breast cancer 1, early onset
BRCA2: breast cancer 1, early onset
IMOAB: Aldenora Bello Maranhense Institute of Oncology
CACON: Reference Center of High Complexity in Oncology
